# Succinate dehydrogenase deficient gastrointestinal stromal tumor in a three month old boy with a fatal clinical course: a case report and review of literature

**DOI:** 10.1186/s13000-021-01077-4

**Published:** 2021-02-21

**Authors:** Bei-Bei Lv, Jia-Mei Li, Zhi-Gang Yao, Xian-Kui Cheng, Fu-Xin Ren, Wen-Jing Su, Ye-Jun Qin, Zhou Wang, Zhi-xin Cao

**Affiliations:** 1 Department of Pathology, Shandong provincial hospital affiliated to Shandong First Medical University, No. 324 Jing Wu Road, Jinan, 250021 Shandong Province China; 2 Department of Pathology, Shandong Provincial Hospital affiliated to Shandong University, Jinan, 250021 Shandong Province China; 3 Shandong Medical Imaging Research Institute, Shandong University, Jinan, 250021 Shandong Province China

**Keywords:** SDHB, GIST, Infant, Genetic characteristics, Case report

## Abstract

**Background:**

Succinate dehydrogenase deficient gastrointestinal stromal tumors (SDH-deficient GISTs), which lack KIT or PDGFRA mutations demonstrate unique clinical and pathological features, and they respond poorly to standard targeted therapy. We herein present a novel case of SDH-deficient GIST in a three-month-old infant’s colon mesentery, and he is the youngest patientto date.

**Case presentation:**

The infantpresented with complaints of blood in the stool. CT showed a 6.3 × 4.6 cm mass in the left lower retroperitoneal. Complete resection of tumor and segmental bowel resection was performed without regional lymphadenectomy. Histologically, tumor cells were distinctive in their multinodular colon wall involvement with interspersed tracts of colon wall smooth muscle. The tumor was composed mainly of epithelioid cells. Immunohistochemically, the tumor cells were positive for Vim, CD117, PDGFR, while negative for SDHB. Mutational analysis showed a synonymous mutation for SDHB and wild-type for KIT and PDGFRA. Two months after surgery, metastases were found and Imatinib was administered. Unfortunately, the disease continued to progress, and the infant died 5 months after surgery.

**Conclusions:**

SDH-deficient GISTs comprise a subgroup of a relatively rare tumor type and show a number of clinically and biologically unique features, especially for infants. It is of great importance to developing new therapeutic targets and novel specific drugs.

## Background

Gastrointestinal stromal tumors (GISTs) are the most common mesenchymal tumors of the gastrointestinal tract which have been recognized as genetically and biologically heterogeneous tumors. Most GISTs harbor activating mutations of KIT or platelet-derived growth factor receptor alpha (PDGFRA) [1]. Approximately 15% of GISTs in adults and more than 90% of pediatric GISTs lack these tyrosine kinase mutations and they are generally classified as “wildtype” (WT) GISTs [1, 2]. Among them, succinate dehydrogenase (SDH)-deficient GISTs, which are associated with SDH deficiency by immunohistochemistry (IHC), are the largest group [3]. SDH-deficient GISTs occur exclusively in the stomach and are characterized by a distinctive multinodular/plexiform architecture and epithelioid or mixed epithelioid and spindle cell morphology [4, 5]. A small subset of patients with SDH-deficient GIST accompanies with Carney-Stratakis syndrome or Carney triad [6]. We herein present a novel case of SDH-deficient GIST in a three-month-old infant’s colon mesentery, exhibiting all the clinical, morphological, immunohistochemical, and genetic characteristics of this rare tumor, followed by a brief discussion on this rare entity. To our knowledge, this is the youngest case reported to date.

## Case presentation

**Fig. 1:**
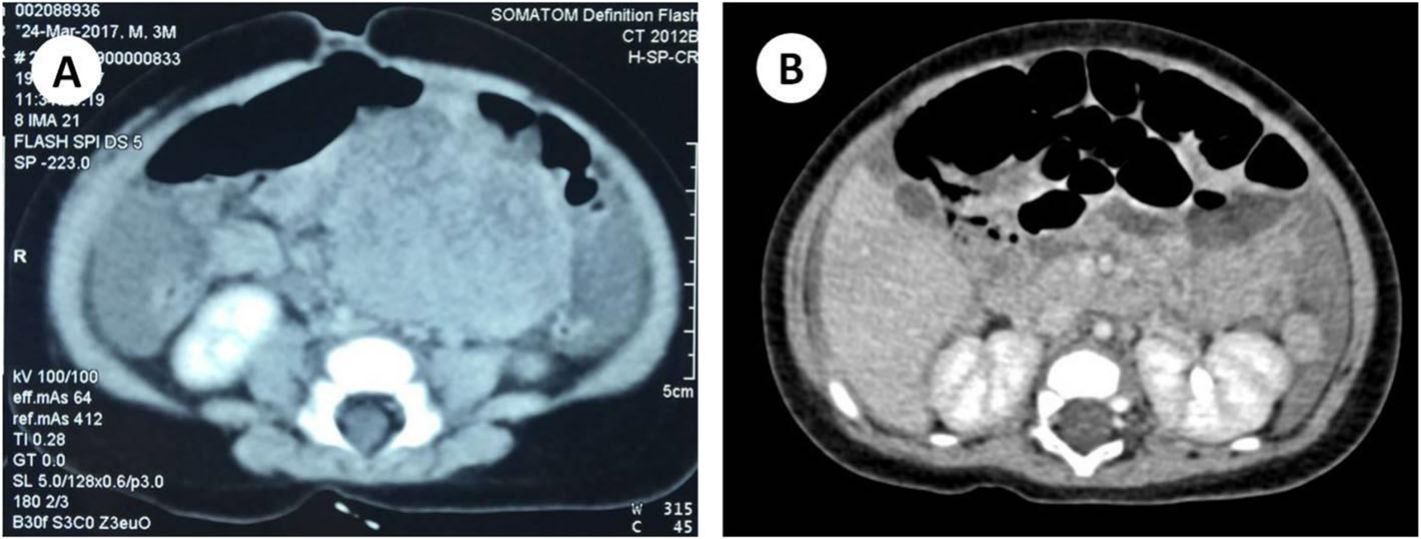
Radiologic findings of the horizontal views. **a**: Enhanced CT showing a 6.3 × 4.6 cm mass in the left lower retroperitoneal with a clear boarder. **b**: Enhanced CT findings suggested that multiple metastases were seen in retroperitoneal

A three-month-old baby boy presented with complaints of blood in the stool with a duration of more than half a month. On clinical examination, B-ultrasound results suggested that solid hypoechoic mass was detected in the left lower abdomen, the size was about 6.9 × 5.0 × 3.9 cm. Enhanced Computed tomography (CT) showed a 6.3 × 4.6 cm mass in the left lower retroperitoneal (Fig. 1a). The border is clear, showing uneven reinforcement. No other physical abnormalities were found. Blood routine examination revealed that hemoglobin was 63 g/L and the red blood cell count was 2.15 × 10^12^/L. The baby’s parents and elder sister are in good health and no related neoplastic lesions were found.

Complete resection of the tumor and segmental bowel resection was performed without regional lymphadenectomy in the pediatric surgery department. The tumor located in the left mesentery with invasion into the colon and sigmoid junction. Adjuvant anti-cancer treatment was not undertaken after the operation on account of bad general condition.

**Fig. 2:**
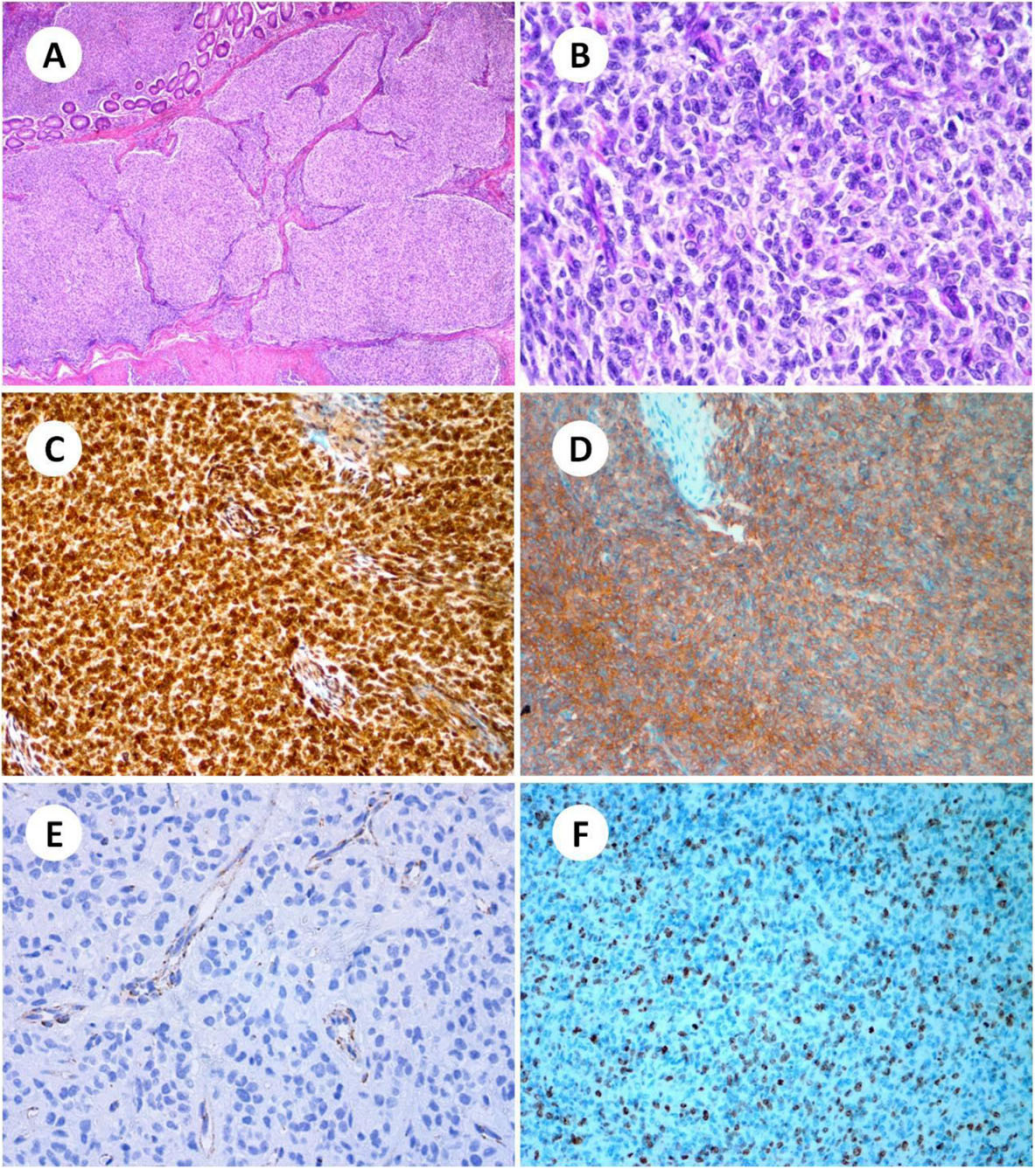
The microscopic features and immunohistochemical stains of the lesion. **a**: Tumors are distinctive in their multinodular colon wall involvement with interspersed tracts of colon wall smooth muscle (H&E, × 100). **b**: The tumor cells have a mainly epithelioid cytology with variably eosinophilic cytoplasm. Mitotic images were easy to be seen (H&E, × 400). **c**: PDGFR was diffusely and strongly positive (× 200). **d**: The tumor cells were positive for CD117 (× 200). **e**: The tumor cells lack SDHB expression, but vascular elements were positive (× 400). **f**: The Ki-67 labelling index (MIB-1 index) reached 30% in the area of greatest concentration (× 200)

Microscopically, under low magnification, tumors were distinctive in their multinodular colon wall involvement with interspersed tracts of colon wall smooth muscle (Fig. 2a), and this is often referred to a “plexiform” pattern as reported before. Under high magnification, the tumor cells have mainly epithelioid cytology with variably eosinophilic cytoplasm. The nucleus was round or oval, and the nucleolus was obvious. Mitotic images were easy to be seen, mitotic count/5 mm (squared) is more than 50. (Fig. 2b). Lymphovascular and nerve invasion were not found under the microscope.

The panel of immune-histochemical stains included CD117, Dog1, CD34, SDHB, S100, SMA, Desmin, Vim, and Ki67. Among them, Vim, CD117, and PDGFR were diffusely and strongly positive (Fig. 2c, d), which supports the diagnosis of gastrointestinal stromal tumors. Besides, the tumor cells lack SDHB expression, but normal intestinal mucosa and vascular elements were positive which verify adequate immunohistochemical detection (Fig. 2e). CD34, Dog1, S100, SMA, Desmin, were negative. The Ki67 labeling index (MIB-1 index) reached 30% in the area of greatest concentration (Fig. 2f).

**Fig. 3:**
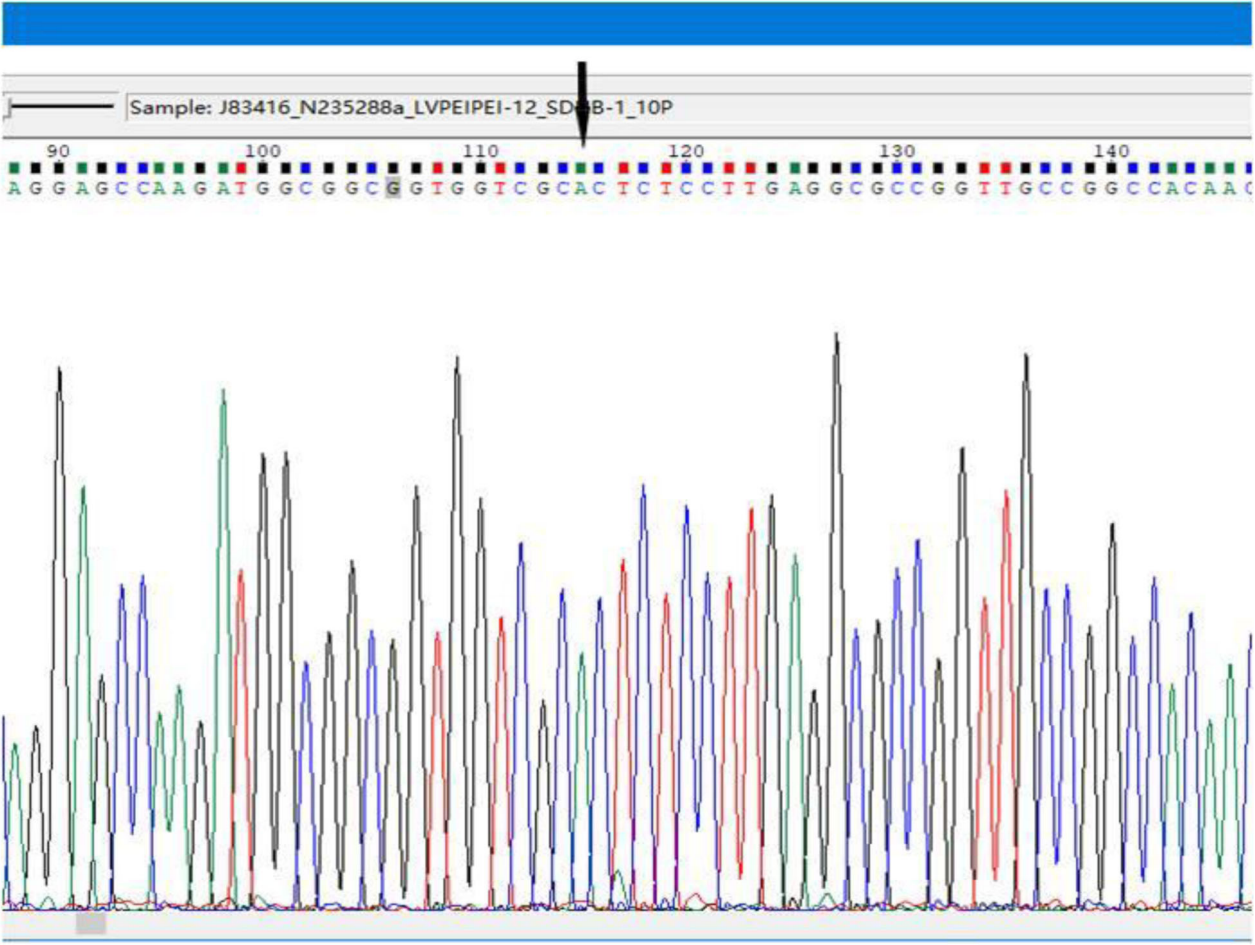
Sequencing results shows mutation at position 169 of exon 1(C-A) (↓ shows mutated loci)

Mutational analysis showed a wild-type for KIT and PDGFRA at the five exons examined (KIT exons 9,11,13,17 and PDGFRA exon 18). In addition, all other targets (Her2、EGFR、RET、ROS1、PI3KCA、ALK、KRAS、NRAS and MET) showed no mutation. However, CCND2 amplification and amino acid missense mutation at position 932 of exon 19 of the PTCH1 gene were detected, which may have a significant impact on gene function. At last, we performed SDHB gene sequencing in Jinan Boshang Biotechnology Co. Ltd. The sequencing of SDHB in tumor showed synonymous mutation at position 169 of exon 1(C-A) (Fig. 3), which may be related to the occurrence and development of this tumor.

Based on these findings, the pathological diagnosis of SDH-deficient GIST was established.

Two months later, the infantwas brought to our hospital again presented with complaints of cough for 2 weeks and diarrhea for 2 days. The results of abdominal color Doppler ultrasound showed that there was much effusion in the abdominal cavity. Abdominal enhanced CT findings suggested that multiple metastases were seen in the peritoneum, mesentery, retroperitoneal, left groin, and right lower abdominal wall (Fig. 1b). Pulmonary CT suggested double lung inflammatory lesions. Anti-infective treatment was carried out in PICU to correct symptomatic and supportive treatment such as anemia. When the condition improved, the infant left our hospital. According to the advice of Zhongshan First Affiliated Hospital, Imatinib (100 mg‚ once daily) was administered. Because of severe diarrhea, the medicine was withdrawal after 3 weeks. Unfortunately, the disease continued to progress, and the infant died 5 months after surgery.

## Discussion

SDH-deficient GISTs represent the largest proportion of WT GISTs that lack KIT or PDGFRA mutations. The true frequency of SDH-deficient GISTs was reported to be approximately 7.4 to 7.7% [6–8]. This group encompasses most pediatric GISTs and two previously described syndromes: Carney-Stratakis syndrome and Carney triad.

Succinate dehydrogenase (SDH) is an enzyme complex composed of four protein subunits (SDHA, SDHB, SDHC, and SDHD). This complex acts at the interphase of the tricarboxylic acid cycle and electron transport chain. The SDH-complex participates in the Krebs cycle with subunit A (SDHA) being the catalytic unit responsible for the conversion of succinate to fumarate. Subunit B (SDHB) is an iron-sulfur protein that participates in the electron transport chain for the oxidation of ubiquinone to ubiquinol. Subunits C and D (SDHC and SDHD) are membrane-anchoring subunits. Remarkably, immunohistochemistry for SDHB becomes negative whenever there is bi-allelic inactivation of any component of SDH, which is very rare in the absence of syndromic disease [9]. Loss of SDHB, as tested by immunohistochemistry, is the most practical way to identify SDH-deficient tumors [3, 7]. Loss of function of the succinate dehydrogenase complex characterizes a rare group of human tumors including some gastrointestinal stromal tumors, paragangliomas, renal carcinomas, and pituitary adenomas. They can all be characterized as SDH-deficient tumors [9, 10].

SDH-deficient GISTs demonstrate unique clinical and pathological features, including an exclusively gastric location, absence of KIT or PDGFRA mutations, typically showed plexiform muscularis propria involvement, and epithelioid hypercellular morphology. Based on current experience, SDH-deficient GISTs occur mainly in the stomach and were more likely to occur in younger, female patients. In this report, we showed a colon mesentery tumor case in a three-month-old infant, which was the youngest patient also in an unusual location reported to date [3, 7, 8, 11]. Malignant tumors in infants and young children may represent a different subgroup because of their unique clinical features and biological behavior. The cause of the tumor is unclear. Whether it is associated with embryonic development needs more cases to study.

SDH-deficient GISTs are characterized by a distinctive multinodular/ plexiform architecture and epithelioid or mixed epithelioid and spindle cell morphology [4]. In our case, the “plexiform” pattern is apparent, and the tumor cells have mainly epithelioid cytology, which is consistent with the reports before and support for the diagnosis. Not surprisingly, IHC examinations for SDH-deficient GISTs showed positivity for CD117, CD34, DOG-1 and PDGFR, and negativity for SMA and S-100. Loss of SDHB expression is a consistent feature of SDH-deficient GISTs. The most important molecular change of SDH-deficient GISTs is SDH mutations followed by SDHC promoter hypermethylation [3, 12]. SDH mutations are often germline and most commonly A (about 30%), and B, C, or D (together 20%) [3, 6, 13]. It is unknown how mutations in the various SDH subunits may differentially regulate tumor biology. Patients with alterations of the SDHC gene may be less likely to develop distant metastases [6]. Besides, another feature of SDH-deficient GISTs is overexpression of insulin-like growth factor 1 receptor (IGF1R) gene, possibly by gene amplification. Chou A et al [14] used immunohistochemistry suggested that IGF1R is overexpressed in 100% of SDH-deficient GISTs but never in non-SDH deficient GISTs. In our case, the sequencing of SDHB showed synonymous mutation at position 169 of exon 1(C-A) and the mutation site agrees with the results of the previous study [15]. Although amino acid has not changed, synonymous mutation frequently acts as driver mutations in human cancers. Fran Supek et al. present robust statistical evidence in an analysis of > 3000 cancer exomes and > 300 cancer genomes that synonymous mutations in exons may act through diverse molecular mechanisms, and are often associated with changes in splicing [16]. Marc Bennedbæk et al. identified SDHB, SDHC, SDHD germline variants in Danish pheochromocytoma/paraganglioma patients and founded that all three SDHB missense variants were predicted as pathogenic. In silico splicing analysis indicates that this change could affect the splicing of exon 5 [17]. Therefore, we speculate synonymous mutation of SDHB in our study may be pathogenic through the same mechanism. Besides, the tumor has an SDHA or SDHC alteration is also possible.

In addition, CCND2 amplification and amino acid missense mutation at position 932 of exon 19 of the PTCH1 gene was found. CCND2 belongs to the highly conserved cyclin family, forms a complex with CDK4 or CDK6, and functions as a regulatory subunit of the complex, whose activity is required for cell cycle G1/S transition. CCND2 amplification has been previously reported in GIST [18]. PTCH1 encodes a member of the patched family of proteins and a component of the hedgehog signaling pathway, which is important in embryonic development and tumorigenesis [19]. Whether the changes in these two genes are related to the tumor needs more cases for further study.

SDH-deficient GISTs do not seem to have a marked tendency for familial occurrence, as compared with SDH mutation syndrome associated paragangliomas that show familial occurrence. Due to the rarity of SDH-deficient GISTs, treatment experience is limited, especially for pediatric patients. Complete surgical removal of the primary tumor and locoregional (omental or nodal) metastases should be performed whenever possible. There is no uniform about the adjuvant treatment of patients with SDH-deficient GISTs to date. Traditional cytotoxic chemotherapy is generally ineffective for SDH-deficient GISTs, as it is for KIT/PDGFRA mutant GISTs. SDH-deficient GISTs respond poorly to standard targeted therapy such as tyrosine kinase inhibitor drugs, neither the first line inhibitor imatinib mesylate nor a second line multikinase inhibitor sunitinib malate, although the stable disease has been observed in some cases [6, 8, 20]. Newer tyrosine kinase inhibitor drugs are potentially usable in SDH-deficient GISTs include regorafenib, nilotinib, and sorafenib [21]. Better molecular and clinical characterization could improve management.

Conventional risk stratification fails to predict the progression of SDH-deficient GISTs [6]. SDH deficient GISTs run a relatively indolent course despite their frequent lymph node or distant metastasis. Follow-up data shows some patients survived for 10 ~ 17 years after peritoneal metastases [7]. However, our case only survived for 5 months after surgery, which may be related to the unique nature of infant and young child malignancies. This case reminds us that such tumors should be vigilant and it is necessary to collect more cases to study the appropriate treatment.

## Conclusions

Consequently, SDH-deficient GISTs comprise a subgroup of a relatively rare tumor type and show some clinically and biologically unique features. It is of great importance to developing new therapeutic targets and novel specific drugs. The priority for further research in this molecular subtype is more extensive sequencing with methods such as whole-exome sequencing, RNA sequencing, and whole-genome sequencing to discover novel genomic events affecting kinases that could suggest therapeutic vulnerabilities. We reported the youngest case of SDH-deficient GIST arising in colon mesentery, and reviewed the relevant literature to make a deeper understanding of the disease, and provide useful parameters for further gene therapy.

## Data Availability

All data generated or analyzed during this case are included within the article.

## Abbreviations

GIST: Gastrointestinal stromal tumors
SDH: Succinate dehydrogenase
CT: Computed tomography
IHC: immunohistochemistry
WT: wildtype
